# *Myrciaria tenella* (DC.) O. Berg (Myrtaceae) Leaves as a Source of Antioxidant Compounds

**DOI:** 10.3390/antiox8080310

**Published:** 2019-08-15

**Authors:** Ana Raquel Carneiro Ribeiro, Maria Lúcia da Silva Cordeiro, Larissa Marina Pereira Silva, Cesar Orlando Munoz Cadavid, Ricardo Basílio de Oliveira Caland, Marília Medeiros Fernandes-Negreiros, Moacir Fernandes Queiroz, Jefferson da Silva Barbosa, Cicero Flavio Soares Aragão, Silvana Maria Zucolotto, Riva de Paula Oliveira, Hugo Alexandre Oliveira Rocha, Kátia Castanho Scortecci

**Affiliations:** 1 Programa de Pós-Graduação em Bioquímica, Centro de Biociências, Universidade Federal do Rio Grande do Norte-UFRN, Centro de Biociências, Natal, Rio Grande do Norte (RN) 59.072-970, Brazil; 2 Laboratório de Transformação de Plantas e Análise de Microscopia (LTPAM), Departamento de Biologia Celular e Genética, Centro de Biociências, UFRN, Natal-RN 59.072-970, Brazil; 3 Laboratório de Produtos Naturais (PNBio), Departamento de Farmácia, Centro da Saúde, UFRN, Natal-RN 59012-570, Brazil; 4 Laboratório de Controle de Qualidade de Medicamentos (LCQMed), Departamento de Farmácia, Centro da Saúde, UFRN, Natal-RN 59012-570, Brazil; 5 Laboratório de Genética Bioquímica (LGB), Departamento de Biologia Celular e Genética, Centro de Biociências, UFRN, Natal-RN 59.072-970, Brazil; 6 Instituto Federal de Educação, Ciência e Tecnologia do Piauí – IFPI, Terezina-PI 64000.00, Brazil; 7 Laboratório de Biotecnologia de Polímeros Naturais (BIOPOL), Departamento de Bioquímica, Centro de Biociências, UFRN, Natal-RN 59.072-970, Brazil

**Keywords:** medicinal plants, cambuí, Myrtaceae, phenolic compounds, antioxidant, antiproliferative, tumor cell line, *Caenorhabditis elegans*

## Abstract

*Myrciaria* species are widely studied to identify their chemical composition and evaluate their biological activity. Since evidence supporting the potential antioxidant and antiproliferative activity of *Myrciaria tenella* is lacking, the aim of this work was to evaluate these activities in six different leaf extracts: hexane (CHE), chloroform (CCE), ethanolic (CEE), methanolic (CME), aqueous final (CFAE), and only aqueous (CAE). The presence of phenolic compounds, tannin, saponin, and ursolic acid was determined by thin layer chromatography (TLC). CEE, CME, and CFAE showed in vitro antioxidant activity at the initiation, propagation, and termination stages of oxidative damage. Moreover, no toxicity was observed in the 3T3 non-cancerous cell line. On the other hand, all extracts promoted cell death in the tumor cell lines human cervical adenocarcinoma cell line (HeLa) and human stomach gastric adenocarcinoma cell line (AGS). Based on these results, the effect of CEE on the AGS cell line was analyzed using flow cytometry, and necrosis and late apoptosis were observed. Finally, the *Caenorhabditis elegans* model showed that CEE was able to reduce the basal reactive oxygen species (ROS) level. Ultra-performance liquid chromatography (UPLC) analysis showed rutin as the major compound in CEE. Therefore, *Myrciaria tenella* fresh leaves may be potential sources of molecules possessing antioxidant and antiproliferative activities.

## 1. Introduction

The use of medicinal plants is an ancient practice in traditional medicine. Furthermore, in the last few decades, medicinal plants have received attention in research in order to discover new bioactive molecules and sources of dietary supplementation [1]. Therefore, the plant secondary metabolites are considered a complex mixture and may be used to treat different diseases, since they may possess antioxidant, antimicrobial, antiproliferative, antifungal, antiparasitic, immunomodulatory, and other activities [2,3,4]. In addition, these new bioactive molecules may be used directly, or form the basis for the development of new drugs with minor modifications, in order to be improve the effectiveness or reduce the toxicity.

Furthermore, the imbalance between reactive oxygen species (ROS) production and degradation has been associated with diseases such as Parkinson’s disease, Alzheimer’s disease, diabetes, heart disease, and cancer [5,6]. Therefore, the antioxidant potential of plant compounds has been investigated to be used as drug or dietary supplements [7].

The *Myrciaria* genus belongs to the Myrtaceae family and the following species have been studied: *Myrciaria dúbia* (camu-camu); *Myrciaria floribunda* (camboim); and four species known as “jaboticabeiras”, namely *Myrciaria phytrantha*, *Myrciaria coronata*, *Myrciaria cauliflora,* and *Myrciaria jaboticaba.* From these plants, the tissues studied were the fruits, pulp, seeds, peel, and leaves. The various extracts obtained exhibited antioxidant, antiproliferative, anti-inflammatory, antihypertensive, antimicrobial, antifungal, and gastroprotective activities [4,8,9,10,11]. 

*Myrciaria tenella* (cambuí) is a tree belonging to the Myrtaceae family, present in the Amazon, Caatinga, and Atlantic Forest biomes [12]. It has been verified that this species has anti-inflammatory, antimicrobial, and antimutagenic activities due to the presence of flavonoids, steroids, triterpenoids, and saponins [4,13,14]. However, the antioxidant and antiproliferative activity is yet to be evaluated. In order to analyze this, six extracts were obtained in a serial extraction manner using different solvents. Next, the antioxidant potential was evaluated in vitro and in vivo using the *Caenorhabditis elegans* animal model. In addition, the cytotoxicity of these extracts was evaluated in the 3T3 cell line (non-tumor), as well as the HeLa and AGS tumor cell lines. 

## 2. Materials and Methods 

### 2.1. Plant Material

*Myrciaria tenella* leaves were harvested in February 2016 in Rio do Fogo, Rio Grande do Norte state (RN), Brazil (5°17′3″ S 35°23′9″). This region has tropical weather, with an average temperature of 28.5 °C. One specimen was deposited at the Centro de Biociências herbarium, identified by Dr. Leonardo de Melo Versieux, and received the voucher number 20.383. Furthermore, this work was conducted under authorization of the Brazilian Authorization and Biodiversity Information System (SISBIO) (process number 52084-1).

### 2.2. Leaf Extract Preparation

Fresh leaves were harvested, washed, divided into small pieces, and transferred to a flask in a proportion of 1:10 (plant material:solvent, w/v). Serial extraction was performed using fresh leaves and the following order of solvents (apolar to polar): hexane (Cambuí Hexane Extract (CHE)), chloroform (Cambuí Chloroform Extract (CCE)), ethanol (Cambuí Ethanol Extract (CEE)), methanol (Cambuí Methanol Extract (CME)), and final water (Cambuí Final Aqueous Extract (CFAE)). The flask containing the fresh leaves and hexane was covered with an aluminum foil to protect against light and shaken at 50 rpm for 24 h. Then, the extract was filtered on a Whatman paper No. 1 and dried using a rotary evaporator at 45 °C. After each extract was filtered, the leaves were transferred to the flask again, and the next solvent was added (serial extraction). Additionally, an extract only with water (Cambuí Aqueous Extract (CAE)) was prepared. In this case, fresh leaves (100 g) were shaken in water at 50 rpm for 24 h. Next, the material was filtered as described earlier. These extracts were weighed and then suspended in dimethyl sulfoxide (DMSO) to a final concentration of 10 mg/mL (stock extract).

### 2.3. Total Sugar, Protein, and Phenolic Compounds

Total phenolic compounds were determined by using Folin–Ciocalteu’s colorimetric method with gallic acid [15]. Protein was measured using the Bradford [16] protocol. The amount of sugar was measured by the phenol-H_2_SO_4_ method using D-glucose (Sigma-Aldrich) as a standard [17].

### 2.4. Phytochemistry Screening by Thin Layer Chromatography (TLC)

The thin layer chromatography (TLC) analysis was performed in duplicate using glass plates with silica gel F_254_. The different mobile phases used to analyze the chemical composition included: (1) ethyl acetate:formicacid:methanol:water (10:0.5:0.6:0.2 - v/v/v/v); (2) ethyl acetate:formicacid:water (8:1:1 - v/v/v); and (3) toluene:methanol:water (9:1:0.1 - v/v/v). The development reagents were sulfuric vanillin, Natural Reagent A 0.5%, and Dragendorff Reagent. The standards used were coumarin, luteolin, ellagic acid, quercetin, kaempferol, catechin, isoorientin, caffeic acid, chlorogenic acid, ursolic acid, and gallic acid. All standards used were purchased from Sigma-Aldrich. The plates were dried and visualized under UV light at 254 nm, and at 365 nm when Natural Reagent A was used. The results were analyzed according to Wagner [18]. 

### 2.5. Antioxidant Activity In Vitro

The six extracts obtained from *M. tenella* were evaluated for antioxidant activity using different in vitro assays, including determination of the total antioxidant capacity (TAC), reducing power, iron chelation, copper chelation, superoxide radical scavenging activity, hydroxyl radical scavenging activity, and 2,2-Diphenyl-1-picrylhydrazyl radical (DPPH). The extract concentrations used were 100, 500, and 1000 µg/mL. The assays were performed as described previously by Nascimento et al. [19].

### 2.6. Cell Viability

For this assay, the effect of the extracts was evaluated for eukaryotic cells: 3T3: a murine fibroblast cell line (NIH/3T3 ATCC^®^ CRL-1658™, Manassas, VA, USA); HeLa: a human cervical adenocarcinoma cell line (HeLa ATCC^®^ CRL-1427™, Manassas, VA, USA); and AGS: a human stomach gastric adenocarcinoma cell line (AGS ATCC ^®^ CRL-1739™). These cell lines were grown in culture flasks with Dulbecco’s Modified Eagle Medium (DMEM, for 3T3 and HeLa cells), and the Ham F12 medium (for AGS cells). The medium was supplemented with fetal bovine serum - FBS (10% v/v) and antibiotics (100 U/mL penicillin and 100 µg/mL streptomycin) and the flasks were maintained in a humidified atmosphere with 5% CO_2_ at 37 °C. For the cell viability assays, cells were plated in 96-well plates at 5 × 10^3^ cell/well until confluence was reached. Next, the extracts were added (EHC, ECC, EEC, EMC, EWFC, and EWC) at final concentrations of 0, 100, 250, 500, or 1000 µg/mL for 24 h at 37 °C with 5% CO_2_. After 24 h, the medium was aspirated and 100 µL of MTT (5 mg/mL) was added. After 4 h at 37 °C and 5% CO_2_, the MTT was solubilized using 100 µL/well of ethanol [19,20]. After 20 min, the absorbance was measured at 570 nm using a spectrophotometer (Biotek Instruments Inc., Winooski, VT, USA). This assay was performed in triplicate for each treatment condition. The MTT reduction was calculated using MTT reduction (%) = (Sample Abs./Control Abs.) x 100.

### 2.7. Annexin V-FITC Apoptotic Activity

The AGS cell line was used to analyze the apoptotic activity using the CEE extract at 1000 μg/mL for 24 h. Cells were grown in 6-well plates at 8 × 10^4^ cell/well [19,20]. Next, cells were labeled with Annexin V and propidium iodide (PI) according to the manufacturer’s instructions taken from the Annexin V Apoptosis Detection kit (Affymetrix ™ and Bioscience Inc., San Diego, CA, USA). After incubation, the fluorescence intensity was measured using a flow cytometer and the data was analyzed in the Flow Jo software.

### 2.8. Antioxidant Activity In Vivo

#### 2.8.1. *Caenorhabditis elegans* Maintenance and Extract Treatment

The N2 (wild type) strain of *C. elegans* was used, which was cultivated in the Nematode Growth Medium (NGM) with *Escherichia coli* OP50 and maintained at 20 °C [21]. Worms were synchronized by treating gravid hermaphrodites with hypochlorite 50% and NaOH 2.5 mM in order to obtain animals at the L1 stage. The CEE was filtered using a 0.22 μm filter and added to the NGM medium at a final concentration of 1 and 10 mg/mL, respectively. The plates were incubated with *E. coli* OP50 for 48 h.

#### 2.8.2. Evaluation of Reactive Oxygen Species (ROS) Production in *C. elegans*

Wild-type animals from the L1 stage were treated with EEC for 48 h at 20 °C. Next, approximately 40 worms per group were transferred to a 96-well plate containing 50 µM H_2_DCFDA (2.7-dichlorofluorescein-diacetate) in PBS. This assay was performed in triplicate. Fluorescence quantification was performed on the VICTOR multiline X3 ™ plate reader (Perkin Elmer, Massachusetts, USA), with excitation at 485 nm and emission at 535 nm. Eight readings were taken, with an interval of 30 min between each reading.

#### 2.8.3. Oxidative Stress Assay

Wild-type animals from the L1 stage were treated with CEE at concentrations of 1 and 10 mg/mL for 48 h at 20 °C. Next, 50 animals per group were transferred to 12-well plates with NGM solid medium and 10 mM tert-butyl hydroperoxide (t-BOOH). A fraction of surviving animals was evaluated up to 27 h using a stereoscopic microscope by probing the animals. This assay was repeated three times with a total number of 150 worms/group.

#### 2.8.4. Effect of CEE on *C. elegans* Development

Approximately 100 L1 larvae were distributed in five NGM plates containing CEE at 1 and 10 mg/mL or with no CEE present. In total, 25 animals from each group were photographed after 48 h of incubation at 20 °C. Body length was measured using the ImageJ software (https://imagej.net/). Experiments were performed in triplicate. 

### 2.9. Ultra-Performance Liquid Chromatography (UPLC)

The CEE extract at 10 mg/mL was filtered using a 0.22 μm membrane (Merck), and 2 μL was injected into the ultra-performance liquid chromatography (UPLC) system (Prominence UPLC-XR^®^, Shimadzu, Kyoto, Japan) with a diode array detector (UPLC-DAD) for the separation of phenolic compounds. The UPLC analysis was formed using a binary analytical pump (LC-20ADXR, Shimadzu,), automatic injector (SIL-20ACXR, Shimadzu), degasser unit (DGU-20A3, Shimadzu), column oven (CTO-20AC, Shimadzu), and photodiode array detector (SPD-M20A, Shimadzu), with the system being controlled by the LC Solution^®^ software. The column used was a Poroshell 120 EC-C18 (50 mm × 4.6 mm i.d., 2.7 μm Agilent^®^). The mobile phase used was a gradient elution with the following solvents: 0.1% formic acid (Pump A), and acetonitrile and 0.1% formic acid (Pump B). The following gradient (35 min as the total time of analysis) was applied: 0 min (99% A and 1% B); 3 min (91% A and 9% B); 19 min (52% A and 48% B); 23 min (5% A and 95% B); 24 min (99% A and 1% B); and 30 min (99% A and 1% B). The flow used in the column was 0.5 mL/min according to Fracassetti et al. [22]. The phenolic compounds were identified by comparing the absorption spectra in the UV-visible (360 nm) region and the retention time of the peak detected in the sample with that of the standard for the substances: rutin, quercetin, pyrogallol, kaempferol, and gallic acid (Sigma-Aldrich). All standards attained a purity greater than 95%. The similarity index (SI) was calculated between the spectra of the peaks present in the extracts and standards, considering values close to 1.0. 

### 2.10. Statistical Analysis

The results are expressed as the mean ± standard deviations. Each assay was performed in triplicate or quintuplet and was repeated 2–3 times. Statistical analysis was performed using GraphPad Prism 6.0 (2014), and the data were submitted to variance analysis (one-way ANOVA) and Tukey’s post-test (*p* < 0.05). 

## 3. Results

### 3.1. Phytochemical Composition

In this study, five extracts from *M. tenella* leaves were obtained in serial order following apolar to polar: CHE, CCE, CEE, CME, and CFAE, and the sixth extract was prepared with only water (CAE). The total phenolic compound (TPC) showed the highest values for CFAE (286.19 ± 10.27 µg), followed by CME (115.85 ± 1.59 µg) and CEE (50.10 ± 0.82 µg), respectively. These three were followed by CAE (22.28 ± 2.73 µg), and CCE (21.23 ± 0.91 µg). Phenolic compounds were not detected in CHE by this method. The TLC analysis only showed the presence of phenolic compounds in CEE, CME, and CFAE; color spots suggestive of terpenes were visualized in almost all the extracts, except in CAE. Furthermore, a spot with a similar color and *Rf* as that of the standard sample of ursolic acid was identified in CHE and CCE samples.

### 3.2. In Vitro Antioxidant Assays

**Figure 1:**
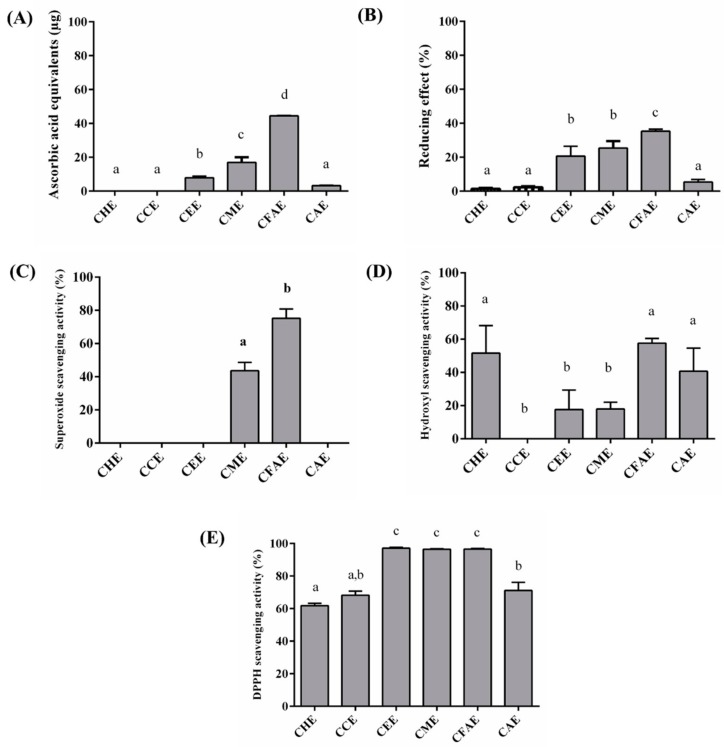
**Antioxidant activity of*Myrciaria tenella* (*M. tenella*) extracts at 100 μg/mL.** (**A**) Total Antioxidant Capacity (TAC). (**B**) Reducing power. (**C**) Superoxide radical scavenging. (**D**) Hydroxyl radical scavenging. (**E**) DPPH assay. For all graphs, CHE (Cambuí Hexane Extract); CCE (Cambuí Chloroform Extract); CEE (Cambuí Ethanol Extract); CME (Cambuí Methanolic Extract); CFAE (Cambuí Final Aqueous Extract); CAE (Cambuí Aqueous Extract). The extracts were tested at a concentration of 100 μg/mL. Eq AA/g: Ascorbic acid equivalents per gram of sample. These assays were performed in triplicate for each extract, and the data were analyzed using ANOVA and Tukey’s test (*p* ≤ 0.05). Different letters (a, b, c) correspond to the significant differences in the statistical analysis.

Phenolic compounds have been associated with different biological functions, such as antioxidant activity, which prevents cell oxidative damage. Six different antioxidant assays were performed: total antioxidant capacity (TAC), reducing power, metal chelation (iron and copper), superoxide radical scavenging, hydroxyl radical scavenging, and DPPH radicals. Figure 1 shows the results obtained for these assays with the extract concentration of 100 μg/mL.

The TAC assay showed that the highest activity was demonstrated by the CFAE (54 μg EqAA/g), followed by CME (20 μg EqAA/g) and CEE (13 μg EqAA/g), respectively (Figure 1A). For the reducing power assay (Figure 1B), the highest activity was observed for the CFAE, followed by CME and CEE. Both CME and CEE had approximately the same effect in the reducing power assay (Figure 1B). The superoxide radical scavenging assay only demonstrated results for CME (43.59 ± 5.05) and CFAE (75.23 ± 5.54%) (Figure 1C). On the contrary, for the hydroxyl radical scavenging assay, the activity was observed for all extracts, except CCE. The activity of CFAE (57.58 ± 2.89) and CHE (51.61 ± 16.55) was approximately similar, and CEE, CME, and CAE showed similar values (40.74 ± 13.86%) (Figure 1D). DPPH activity was detected in all extracts (Figure 1E). The lower values were approximately 60%, and for the polar extracts CEE, CME, and CFAE, the values were approximately 96% (Figure 1E). 

In relation to the metal chelating capacity, different activities were observed. The copper chelating activity was detected for the polar extracts CEE (10% at 100 μg/mL, 51% at 500 μg/mL, and 50% at 1000 μg/mL) and CME (43% for 100 μg/mL, 56% at 500 μg/mL, and 36% for 1000 μg/mL). On the contrary, the highest activity was observed for the CFAE (68% at 100 μg/mL, 56% at 500 μg/mL, and 26% at 1000 μg/mL). Furthermore, the iron chelating activity was only observed for CFAE (36% at 100 μg/mL, 52% at 500 μg/mL, and 43% at 1000 μg/mL).

### 3.3. Cell Viability

**Figure 2:**
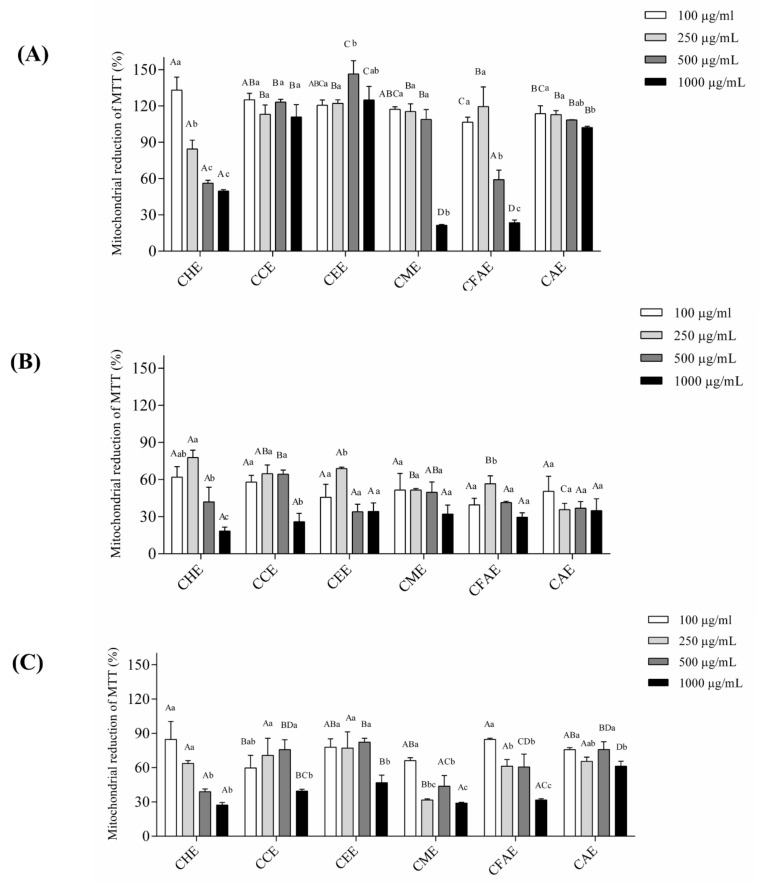
**Cell viability. (A) 3T3 cell line, (B) HeLa cell line, and (C) AGS cell line.** CHE (Cambuí Hexane Extract); CCE (Cambuí Chloroform Extract); CEE (Cambuí Ethanol Extract); CME (Cambuí Methanolic Extract); CFAE (Cambuí Final Aqueous Extract); CAE (Cambuí Aqueous Extract). These assays were performed in triplicate for each extract, and the data were obtained analyzed using ANOVA and Tukey’s test (*p* ≤ 0.05). Different letters (a, b, c) correspond to the significant differences in the statistical analysis.

Considering that these extracts showed an excellent antioxidant activity and that phenolic compounds, terpenes, and saponin were identified, the cytotoxicity of these extracts was evaluated in cell lines. Therefore, the viability was first analyzed in the 3T3 cell line (fibroblast cells from Swiss mice–normal cells) (Figure 2A). These cells were treated with the extracts at different concentrations and the cell viability was evaluated by the MTT method. It may be observed that in general, *M. tenella* extracts were not toxic to the 3T3 cell line. Although cytotoxicity was observed for almost all concentrations of CHE, in the case of CME, cytotoxicity was observed at 1000 μg/mL, and for CFAE, at concentrations of 500 μg/mL and 1000 μg/mL. Based on these results, toxicity was next analyzed in the tumor cell lines HeLa (cervical cancer cells) and AGS (derived from an adenocarcinoma from the stomach). All the extracts reduced the viability of the HeLa cell line (Figure 2B). For example, for the lowest concentration analyzed (100 μg/mL), this reduction ranged from 20.48% to 82.61%, depending on the extract. A reduction in cell viability was reported at 500 μg/mL and 1000 μg/mL for CEE, CFAE, and CAE (Figure 2B). Moreover, a similar effect was observed on AGS cells for all six extracts from *M. tenella* (Figure 2C). Interestingly, it was observed that for the AGS cell line, all extracts, except CME, showed a higher MTT reduction percentage at the highest concentration of 1000 µg/mL (*p* < 0.5) than that at all other concentrations (Figure 2C).

**Figure 3:**
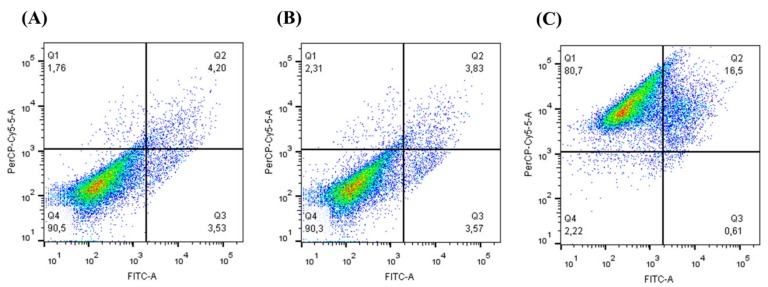
**Annexin V and propidium iodide PI assay of AGS cells exposed to Cambuí Ethanol Extract (CEE).** AGS cells were treated for 24 h with the CEE, stained with annexin V-FITC and PI, and then analyzed by flow cytometry. (**A**) Control: AGS cells stained with annexin V-FITC and PI; (**B**) AGS cells treated with DMSO and stained with annexin V-FITC and PI; (**C**) AGS cells treated with CEE at 1000 µg/mL.

Based on the cell viability results of 3T3, HeLa, and AGS cell lines, the CEE of 1000 μg/mL was chosen to be analyzed in the AGS cell line by flow cytometry (Figure 3). The results showed that DMSO present in the extract did not influence the results (Figure 3B) when compared to those of the control with only annexin and PI (Figure 3A). Moreover, when the AGS cell line was treated with CEE, it was observed that the reduction in cell viability could be associated with a necrosis and late apoptosis mechanism (Figure 3C).

### 3.4. Antioxidant Assays In Vivo Using C. elegans

**Figure 4:**
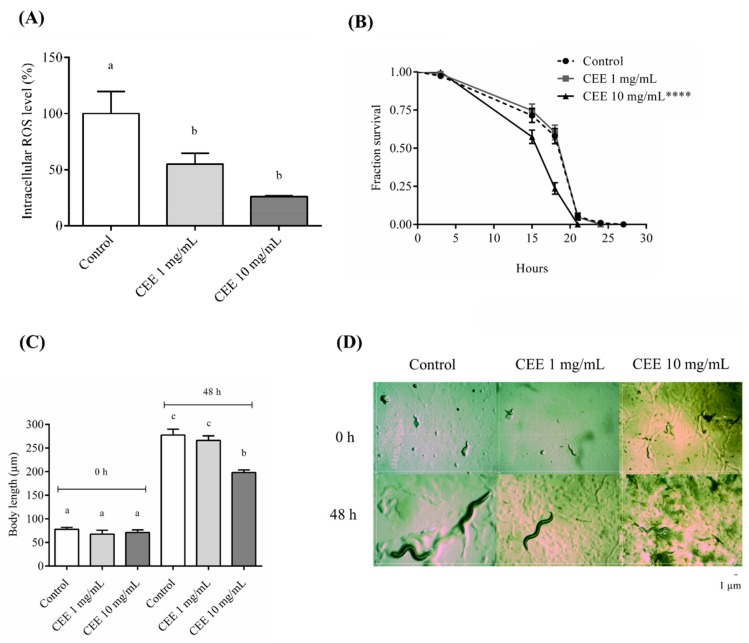
**Cambuí Ethanol Extract(CEE) effect in the*Caenorhabditis elegans*** (***C. elegans*) model.** CEE as an antioxidant in vivo in the *C. elegans* model. (**A**) Levels of reactive oxygen species (ROS) in N2 strains treated with the CEE for 48 h. Three independent experiments were performed, with three replicates per treatment (*n* = 40). (**B**) Survival assay of N2 wild-type animals under oxidative stress conditions (150 animal/group). Worms were treated with CEE at the L1 stage to L4 stage. Survival is observed at 3, 15, 18, 21, 24, and 27 h at 20 °C. The CEE treatment was compared to the control by the Log-rank test (*p* < 0.0001). (**C**) Measurement of the body size of *C. elegans* treated with the CEE after 48 h of exposure. (**D**) Magnified images (magnification 10X) of animals at time 0 and after 48 h of treatment with the CEE. Scale is 1 µm. Different letters correspond to significant differences. The data in the quintuplet for each extract concentration were analyzed by ANOVA and Tukey’s test (*p* ≤ 0.05).

The nematode *C. elegans* was used to evaluate the antioxidant potential of CEE in vivo. In addition to 0.1 mg/mL, a higher CEE concentration of 10 mg/mL was used since the nematode has a cuticle. First, we evaluated whether CEE was able to reduce the intracellular levels of ROS. The results demonstrated that CEE decreased ROS levels to 55.11% ± 9.36% and 26.10% ± 0.75% with 1 mg/mL and 10 mg/mL treatments, respectively, compared to the control (Figure 4A). An evaluation of whether CEE treatment increased the survival of *C. elegans* under oxidative stress conditions induced by t-BOOH was also undertaken. The 1 mg/mL CEE treatment did not alter the *C. elegans* survival compared to the control (Figure 4B). However, the 10 mg/mL CEE concentration reduced *C. elegans* survival under stress conditions (Figure 4B), suggesting that CEE treatment may be toxic or interfere with the development of *C. elegans*. To investigate this possibility, we analyzed the body length of L1 and L4 animals. We observed that 1 mg/mL CEE treatment did not cause any significant change in the worm development when compared to the control treatment. However, at 10 mg/mL CEE, a reduction of 28.7% was observed when compared to the control (Figure 4C,D).

### 3.5. UPLC Analysis

**Figure 5:**
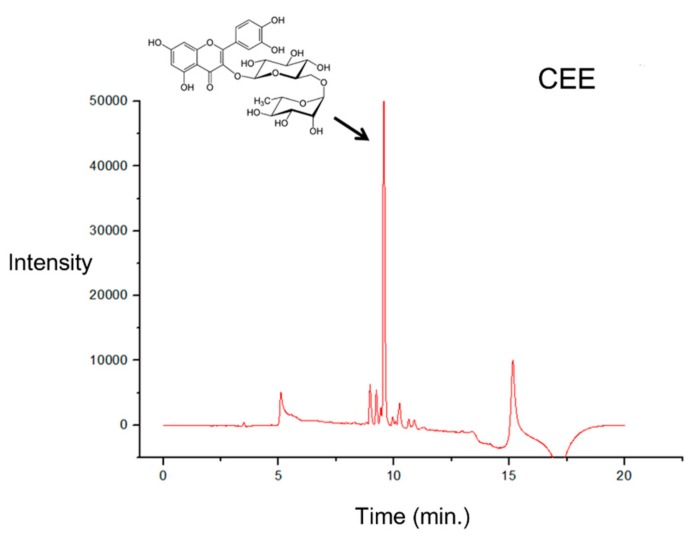
**Chromatogram of the Cambuí Ethanol Extract(CEE) using theultra-performance liquid chromatography** (**UPLC) approach.** The detection wavelength was set at 360 nm. The x-axis corresponds to the retention times (min) of each peak. The peak absorption spectra present in the CEE (Tr: 9.5) were compared with the rutin pattern (TR: 9.3), obtaining a similarity index (IS) above 0.9.

Based on the results demonstrating the CEE-related antioxidant activity in vitro and in vivo, cell viability, and cell antiproliferative activity, CEE was further analyzed by the UPLC in order to characterize the chromatographic profiles of the extracts. Through the maximum absorption of the UV spectrum (256 to 350 nm) and retention time (9.5 min) when compared with the standard of rutin, the presence of the flavonoid rutin was identified as the major compound (Figure 5). When the rutin solution was added in the extract solution (1:1, v/v), an increase in the peak area at 9.5 min was observed.

## 4. Discussion

The study on medicinal plants has intensified the search for new molecules exhibiting different biological activities as these plant extracts are a complex mixture of compounds. The antioxidant effect is considered crucial, as the imbalance of ROS has been associated with a number of diseases, such as cancer, arteriosclerosis, diabetes, Parkinson’s disease, and Alzheimer’s disease [2,23]. 

In the case of *Myrciaria tenella,* only a few studies have evaluated its biological activity. Hence, we performed a serial extraction using fresh leaves and non-polar to polar solvents to obtain the corresponding series of extracts: hexane (CHE), chloroform (CCE), ethanolic (CEE), methanolic (CME), aqueous final (CFAE), and only aqueous (CAE). The premise of serial extraction is the separation of different molecules according to the solvent polarity. In this study, it was observed that CEE, CME, and CFAE were more effective in extracting phenolic compounds, probably due to the polar chemical nature of the solvents used, ideal for the extraction of phenolic compounds [24]. 

The presence of phenolic compounds in CEE, CME, and CFAE was confirmed by TLC. In addition, the presence of terpenes was identified in all extracts, except CAE, and the triterpenoid ursolic acid was identified in CHE and CCE, as well as saponins in CEE, CME, and CFAE. These results corroborate the data of Alice et al. [13] and Schneider et al. [25], who detected phenolic compounds, triterpenoids, and saponins in *M. tenella* leaves using an ethanolic extract. Ursolic acid is a triterpene that has demonstrated anti-inflammatory, antioxidant, and antiproliferative activities [26,27,28,29], while saponins may exert a cytotoxic action, probably by membrane interaction [30]. Moreover, the presence of phenolic compounds (anthocyanins, flavonoids, and tannins) has been identified in *Myrciaria cauliflora, Myrciaria dubia, Myrciaria vexator,* and *Myrciaria floribunda* [8,9,11,22,31,32]. 

The antioxidant activity has three different steps (initiation, propagation, and termination) conducted through different mechanisms [33]; here, the antioxidant activity was evaluated using six assays. In the case of the total antioxidant capacity (TAC), CFAE reported the highest activity, followed by CME and CEE, whereas in the evaluation of the reducing power and hydroxyl scavenging, a greater percentage of activity was observed for the polar extracts. 

Furthermore, CFAE reported iron chelating activity. Iron activity is important for patients with thalassemia, and iron chelation has been observed to be important for the treatment of neurodegenerative diseases, having a moderate effect in 80% of the patients [34]. The copper chelating activity was observed in CME, CEE, and CFAE. A copper imbalance in organisms has been associated with neurodegenerative disorders such as Wilson’s disease, Menkes disease, Alzheimer’s disease, and Parkinson’s disease [35]. 

The DPPH assay was associated with the oxidative damage at the termination stage. All the extracts evaluated presented an activity greater than 60%. Furthermore, CEE, CME, and CFAE had superior activity (almost 90%) compared to the other extracts. Similar results were observed for the DPPH assay in *Myrciaria cauliflora* [36]. Our data suggest that CEE, CME, and CFAE are promising extracts due to their antioxidant activity.

Moreover, oxidative stress may promote DNA lesions that can lead to the propagation of tumor cells [37]. Our data showed that, except for the CHE, the other extracts did not show toxicity in the 3T3 non-tumor cell line (murine fibroblasts), while all extracts showed antiproliferative activity in HeLa (cervical cancer cells) and AGS tumor cell lines (adenocarcinoma cells from the stomach). Wang et al. [37] showed that the *M. cauliflora* aqueous extract from seeds at concentrations of 1 μg/mL and 10 μg/mL reduced the viability of HSC-3 cells (from an oral carcinoma). Tietbohl et al. [10] showed that the *M. floribunda* leaf extract at a concentration of 250 μg/mL had an antiproliferative effect in different tumor cell lines. In this study, a reduction in viability was observed for *M. tenella* in HeLa and AGS cell lines. This antiproliferative activity may be associated with the phytochemical composition, including phenolic compounds, triterpenoids, saponins, and tannins [38]. Moreover, the flow cytometry data showed that the antiproliferative activity of the CEE was related to necrosis and late apoptosis. Necrosis or necroptosis has been associated with programmed cell death regulated by different signals, as well as by caspase 8 inactivation or receptor-interacting protein kinases (RIPKs) activation [39,40]. Therefore, the CEE from *M. tenella* may induce different signal-transduction pathways and specific cell death mediators to promote necrosis. Additionally, the UPLC data reported that the CEE had a peak corresponding to rutin, a flavonoid commonly found in dark-colored fruits that has antioxidant activity and is known to promote different biological activities [41,42,43]. Alajmi et al. [44] showed, by protein modeling, that rutin may interact with DNA topoisomerase II, which could be a target for anticancer drugs [44]. Therefore, the presence of rutin and other molecules in CEE could play a role in promoting the necrosis and late apoptosis observed in the AGS cell line. 

The data obtained with the *C. elegans* model reinforced the antioxidant in vitro results, as the CEE was able to reduce the basal ROS. This antioxidant effect may be attributed to the presence of rutin in the extract [45]. Kampkotter et al. [46] showed that treatment with 100 µM of rutin reduced ROS levels by 15% in *C. elegans.* Our results showed that CEE treatment reduced ROS production from 45 to 75%. This result may be a synergistic effect, as the CEE extract is a mixture of molecules including phenolic compounds, tannin, saponin, and rutin.

Despite its antioxidant activity in vivo, the CEE did not increase *C. elegans* resistance against oxidative stress. At 1 mg/mL of CEE, the survival of the worm was not significantly different from untreated animals. Moreover, animals treated with 10 mg/mL had reduced survival under stress, and their body length indicated that the CEE at this concentration is toxic to the worms. Our results showed that the CEE at 10 mg/mL affected the worm’s life span and development, raising some hypotheses: firstly, since the CEE is a mixture of molecules including rutin, which has antimicrobial activity, this may affect the OP50 *E. coli* growth and consequently the *C. elegans* ingestion [4,44,47]; secondly, rutin or tannins present in the CEE may react with the proteins or glycoproteins present in the *C. elegans* cuticle and affect the development and life span of the worm [48,49]; and lastly, a third scenario could postulate that the CEE antioxidant activity at 10 mg/mL reduces ROS production to a deleterious level, creating an unbalanced REDOX state in the worm. These hypotheses may explain why the CEE was able to affect stress resistance and worm development, despite reducing the basal ROS level. Moreover, it was interesting to observe that the CEE did not have any toxic effect on the non-cancerous 3T3 cell line, but only promoted necrosis and late apoptosis in the tumor cells HeLa and AGS (stomach cell line). Therefore, these plant extracts have the potential to be used as therapeutic agents or as dietary supplements.

## 5. Conclusions

*Myrciaria tenella* leaves are a potential source of several classes of secondary metabolites, such as terpenoids, phenolic compounds, flavonoids, and tannins, with known antioxidant effects. Therefore, the identification of the chemical components of these extracts, also in terms of minor components, will be our goal in future investigations. CEE and CFAE were highlighted to have antioxidant potential in vitro. In *C. elegans*, CEE demonstrated potential antioxidant activity and UPLC analysis showed rutin as CEE’s major compound. Moreover, the extracts were not toxic to the non-tumor 3T3 cell line, but showed antiproliferative effects on HeLa and AGS cell lines. Taken together, CEE shows excellent promise to be further investigated, and it may be considered a sustainable alternative for the production of herbal products.
